# Artificial intelligence-enhanced detection of subclinical coronary artery disease in athletes: diagnostic performance and limitations

**DOI:** 10.1007/s10554-024-03256-y

**Published:** 2024-10-07

**Authors:** Jens Kübler, Jan M. Brendel, Thomas Küstner, Jonathan Walterspiel, Florian Hagen, Jean-François Paul, Konstantin Nikolaou, Sebastian Gassenmaier, Ilias Tsiflikas, Christof Burgstahler, Simon Greulich, Moritz T. Winkelmann, Patrick Krumm

**Affiliations:** 1 Department of Radiology, Diagnostic and Interventional Radiology, University of Tübingen, 72076 Tübingen, Germany; 2 Department of Radiology, Institut Mutualiste Montsouris, Cardiac Imaging, 75014 Paris, France; 3 Spimed-AI, 75014 Paris, France; 4 Department of Internal Medicine III, Cardiology and Angiology, University of Tübingen, 72076 Tübingen, Germany; 5 Department of Internal Medicine V, Sports Medicine, University of Tübingen, Tübingen, Germany

**Keywords:** Coronary artery disease, Coronary computed tomography angiography, Artificial intelligence, Fractional flow reserve, Diagnostic accuracy, Marathon runners

## Abstract

**Purpose:**

This study evaluates the diagnostic performance of artificial intelligence (AI)-based coronary computed tomography angiography (CCTA) for detecting coronary artery disease (CAD) and assessing fractional flow reserve (FFR) in asymptomatic male marathon runners.

**Material and methods:**

We prospectively recruited 100 asymptomatic male marathon runners over the age of 45 for CAD screening. CCTA was analyzed using AI models (CorEx and Spimed-AI) on a local server. The models focused on detecting significant CAD (≥ 50% diameter stenosis, CAD-RADS 3, 4, or 5) and distinguishing hemodynamically significant stenosis (FFR ≤ 0.8) from non-significant stenosis (FFR > 0.8). Statistical analysis included sensitivity, specificity, positive predictive value (PPV), negative predictive value (NPV), and accuracy.

**Results:**

The AI model demonstrated high sensitivity, with 91.2% for any CAD and 100% for significant CAD, and high NPV, with 92.7% for any CAD and 100% for significant CAD. The diagnostic accuracy was 73.4% for any CAD and 90.4% for significant CAD. However, the PPV was lower, particularly for significant CAD (25.0%), indicating a higher incidence of false positives.

**Conclusion:**

AI-enhanced CCTA is a valuable non-invasive tool for detecting CAD in asymptomatic, low-risk populations. The AI model exhibited high sensitivity and NPV, particularly for identifying significant stenosis, reinforcing its potential role in screening. However, limitations such as a lower PPV and overestimation of disease indicate that further refinement of AI algorithms is needed to improve specificity. Despite these challenges, AI-based CCTA offers significant promise when integrated with clinical expertise, enhancing diagnostic accuracy and guiding patient management in low-risk groups.

## Introduction

Coronary computed tomography angiography (CCTA) has emerged as a non-invasive technique for assessing coronary artery calcium burden and detecting coronary artery stenosis, offering a high negative predictive value for excluding obstructive stenosis. [1–3]. The incorporation of CT-derived fractional flow reserve (FFR) estimation has gained prominence in evaluating hemodynamically significant stenoses, with invasively determined FFR values below 0.75–0.80 defining stenoses that warrant revascularization [4]. Recent advancements in artificial intelligence (AI)-based models have further enhanced CCTA and FFR assessments. These models provide automated scoring, accelerate workflow, and improve diagnostic accuracy [5].

However, human interpretation of CCTA carries the risk of overdiagnosis, especially in populations with a low prevalence of diseased vessels. Athletes are a unique population who may have a lower prevalence of traditional cardiovascular risk factors, but may still be at risk for coronary artery disease (CAD) due to intense physical activity and genetic predisposition [6]. Understanding and predicting CAD in athletes is crucial for early intervention and prevention of adverse cardiac events. Testing a predictive AI model in this unique population allows for the assessment of its performance in an underrepresented subgroup, providing valuable insights into its applicability and reliability in diverse clinical scenarios, particularly for individuals who may not present typical risk factors for CAD.

The aim of our study was to assess the diagnostic performance of AI-derived CCTA stenosis detection and FFR evaluation of CAD in a low-risk cohort of asymptomatic athletes who prospectively underwent CCTA for screening purposes.

## Material and methods

### Ethics approval

This prospective study has received approval from the institutional review board (processing number 158/2011B01) and the German Federal Office for Radiation Protection (processing number Z5-22,462/2–2011-22), and it complies with the Declaration of Helsinki. Informed consent was obtained from all participants.

### Study subjects

We prospectively recruited 100 asymptomatic male marathon runners over the age of 45 for CAD screening between 2012 and 2014. Participants were excluded if they had known CAD, allergies to iodinated contrast agents, impaired renal function (glomerular filtration rate < 60 mL/min/1.73 m^2^), or hyperthyroidism. The Framingham Risk Score and Agatston calcium score were calculated for each participant [1, 7]. Clinical data and visual CCTA results of study subjects were published previously [8–10].

### Coronary computed tomography angiography acquisition

Examinations were performed using a dual-source CT scanner (Siemens Somatom Definition Flash, Siemens Healthineers). Initially, a scout acquisition was performed with the patient in a supine position. ECG-triggered calcium scoring was then conducted in a cranio-caudal direction using collimation of 2 × 64 × 0.6 mm, a gantry rotation time of 280 ms, a pitch of 3.4, and a tube current of 70 mA with automatic tube current modulation at 120 kV. To determine systemic circulation time, a test bolus of 10 mL of non-ionic iodinated contrast agent (370 mg iodine/mL Ultravist 370, Bayer Healthcare) followed by a 20 mL saline flush at 6 mL/s was administered using a dual-head injector (CT Stellant, Medrad). For CCTA, a dose of 70 mL of contrast agent followed by a saline flush was administered with the same flow parameters. Depending on the heart rate, either a high-pitch (≤ 60 bpm) or a prospective sequential step-and-shoot (> 60 bpm) protocol was used at 60% of the R-R interval. Technical parameters for high-pitch acquisition included collimation of 2 × 64 × 0.6 mm, gantry rotation time of 280 ms, pitch of 3.4, tube current of 350 mA with automatic tube current modulation, and tube voltage of 100 kV. Step-and-shoot acquisition protocol details have been described previously [9]. Reconstruction of images for CCTA was performed with a slice thickness of 0.75 mm (B26f).

### Visual CCTA analysis

Two experienced radiologists (JK and PK) evaluated the CCTA datasets in consensus using axial slices, thin slab maximum intensity projections (MIP), and curved multiplanar reconstructions (cMPR). Coronary artery stenosis was classified according to the Coronary Artery Disease-Reporting and Data System 2.0 (CAD-RADS). Significant CAD was defined as diameter stenosis of 50% or higher (CAD-RADS 3, 4, or 5) [11]. Visual CAD-RADS evaluation was used as the benchmark against which the AI-CCTA model’s diagnostic performance was compared.

### AI-based CCTA analysis

For AI-based CAD evaluation, CorEx (version 2.0; Spimed-AI, Paris, France) was employed. CorEx is a deep learning model designed for the automated analysis of CCTA images [12–14]. cMPR images were automatically generated using CT Cardiac Workflow (Syngo.via, Siemens Healthineers) and processed by two deep learning models, CorEx and Spimed-AI, on a local server. The focus was on detecting significant CAD (≥ 50% diameter stenosis, CAD-RADS 3, 4, or 5). Binary AI-derived fractional flow reserve (FFR) results were used to distinguish hemodynamically significant (FFR ≤ 0.8) from non-significant (FFR > 0.8) CAD. The FFR model also incorporated results from the CAD-RADS model. The AI models were trained using the Inception-v3 convolutional neural network architecture and a dataset of 400 CCTA cases with corresponding FFR values measured during invasive coronary angiography [15].

For this study, the models analyzed curved MPR images at 40-degree intervals along the centerline covering the entire vessel circumference of all three coronary arteries. These images were created using a dedicated workflow (CT Cardiac Workflow syngo.via VB70A HF02, Siemens Healthineers) without manual corrections and exported to a local server hosting the AI models. FFRai was evaluated on a per-vessel basis, predicting hemodynamically significant stenosis (FFRai ≤ 0.8) or non-significant stenosis (FFRai > 0.8).

### Statistical analysis

Data analysis was conducted using proprietary statistical software (SPSS 27, IBM Corp.). Continuous data are expressed as means ± standard deviation unless otherwise specified. Sensitivity, specificity, positive predictive value (PPV), negative predictive value (NPV), and accuracy are presented as percentages with 95% confidence intervals (CI).

## Results

### Subject characteristics

**Fig. 1:**
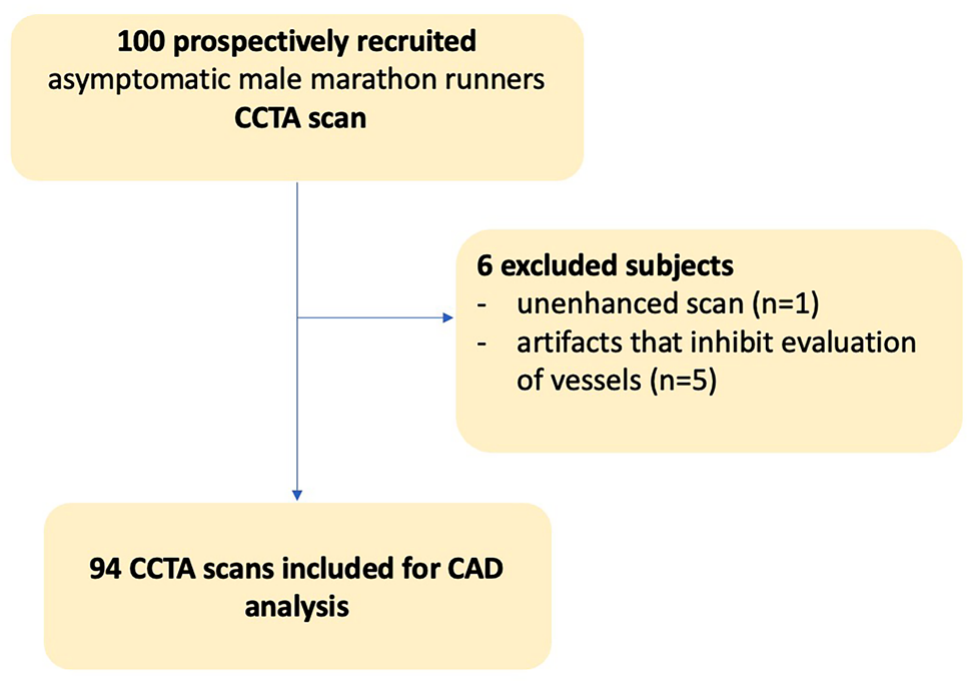
Flowchart of subject inclusion. 100 asymptomatic male marathon runners were recruited for CCTA. Six subjects were excluded due to artifacts that severely impaired evaluation of coronary arteries (n = 5) and one subject due to an unenhanced scan (n = 1). 94 subjects were included for final analysis of coronary artery disease and fractional flow reserve

Of 100 prospectively recruited asymptomatic male marathon runners 94 CCTA scans were included for CAD analysis (Fig. 1). Six subjects were excluded, five due to severe imaging artifacts that inhibited evaluation of coronary arteries, one due to an unenhanced scan.

Detailed characteristics of the 94 included subjects are provided in Table 1.

**Table 1:** Characteristics of 94 subjects who had CCTA scan for CAD analysis

Variables	Values
Age (years)	52.7 ± 6.4 [45–74]
Height (cm)	180 ± 6 [164–195]
Body mass index (kg/m^2^)	23.7 ± 2.3 [18.7–33.1]
Training per week (h)	7.4 ± 5.0 [0–41]
Framingham Score	5.4 ± 3.3 [1.2–20.7]
< 10	87 (92.6%)
10–19	6 (6.4%)
≥ 20	1 (1.1%)
Ca-Score	35.9 ± 100.6 [0–745.8]
*Cardiovascular risk factors*	
Smoking	35 (37.2%)
Arterial hypertension	17 (18.1%)
Diabetes	0
Dyslipidaemia	4 (4.3%)

Quantitative variables are expressed as means ± standard deviations followed by ranges in brackets. Qualitative variables are expressed as raw numbers; numbers in parentheses are percentages.

CAD: Coronary Artery Disease; CCTA: coronary computed tomography angiography.

### Visual and AI-based CAD analysis

**Fig. 2:**
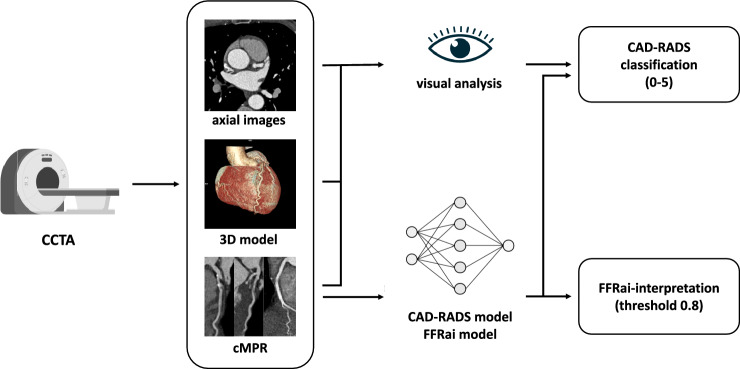
Illustration of coronary artery disease analysis workflow in the study. Coronary CT angiography images were visually interpreted and coronary stenosis was classified according to CAD-RADS (0–5). Curved multiplanar reformations were processed by artificial intelligence-based models (CorEx, Spimed) on a local server. Stenosis was classified according to CAD-RADS (0–5) and FFR was interpreted regarding hemodynamically significant CAD (threshold 0.8)

**Fig. 3:**
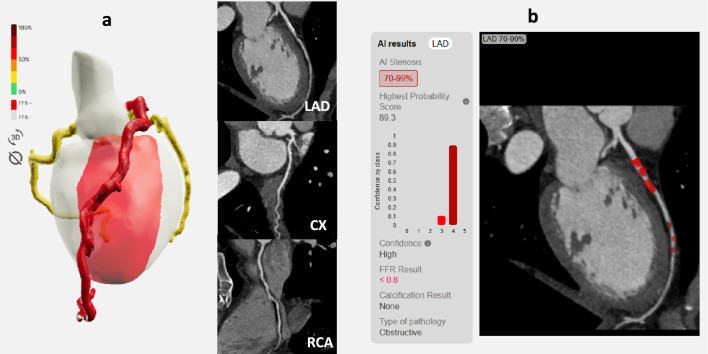
Example of artificial intelligence-based analysis of coronary artery disease in a subject with CAD-RADS 4 stenosis. **A** Coronary artery stenosis was automatically quantified using curved multiplanar reformations. In this example there was a significant stenosis of the LAD (> 70%, CAD-RADS 4). **B** Results of the artificial intelligence evaluation indicated high confidence of the analysis. An additional model calculated fractional flow reserve < 0.8

Details of coronary artery stenosis evaluation are provided in Table 2. The workflow of this study is illustrated in Fig. 2. An example of artificial intelligence-based CAD analysis is provided in Fig. 3.

**Table 2:** Visual and AI-dependent analysis of coronary artery stenosis in 94 subjects

	Visual analysis	AI-analysis
*CAD-RADS analysis*		
*CAD-RADS 0 (0%)*		
all vessels	60 (63.8)	41 (43.6)
LAD	62 (66.0)	46 (48.9)
CX	84 (89.4)	77 (81.9)
RCA	87 (92.6)	67 (71.3)
*CAD-RADS 1 (1–24%)*		
all vessels	19 (20.2)	19 (20.2)
LAD	21 (22.3)	22 (23.4)
CX	6 (6.4)	6 (6.4)
RCA	6 (6.4)	15 (16.0)
*CAD-RADS 2 (25–49%)*		
all vessels	12 (12.8)	22 (23.4)
LAD	8 (8.5)	19 (20.2)
CX	4 (4.3)	7 (7.4)
RCA	1 (1.1)	7 (7.4)
*CAD-RADS 3 (50–69%)*		
all vessels	2 (2.1)	0
LAD	2 (2.1)	0
CX	0	0
RCA	0	0
*CAD-RADS 4 (70–99%)*		
all vessels	1 (1.1)	6 (6.4)
LAD	1 (1.1)	5 (5.3)
CX	0	2 (2.1)
RCA	0	1 (1.1)
*CAD-RADS 5 (100%)*		
all vessels	0	6 (6.4)
LAD	0	2 (2.1)
CX	0	2 (2.1)
RCA	0	4 (4.3)
*Any stenosis (*> *0%)*		
all vessels	34 (36.2)	53 (56.4)
LAD	32 (34.0)	48 (51.1)
CX	10 (10.6)	17 (18.1)
RCA	7 (7.4)	27 (28.7)
*Significant stenosis (*≥ *50%)*		
all vessels	3 (3.2)	12 (12.8)
LAD	3 (3.2)	7 (7.4)
CX	0	4 (4.3)
RCA	0	5 (5.3)

Variables are expressed as raw numbers; numbers in parentheses are percentages.

CAD-RADS: Coronary Artery Disease-Reporting and Data System 2.0; CCTA: coronary computed tomography angiography; CX: circumflex artery; LAD: left anterior descending artery; RCA: right coronary artery.

### Subject level analysis

In visual analysis, on subject level stenosis > 0% in at least one coronary artery was present in 34/94 (36.2%) subjects with a severity of CAD-RADS 1 in 19/94 (20.2%), CAD-RADS 2 in 12/94 (12.8%), CAD-RADS 3 in 2/94 (2.1%), CAD-RADS 4 in 1/94 (1.1%) and CAD-RADS 5 in 0 subjects.

AI-analysis detected stenosis > 0% in 53/94 (56.4%) subjects with a severity of CAD-RADS 1 in 19/94 (20.2%), CAD-RADS 2 in 22/94 (23.4%), CAD-RADS 3 in 0/94, CAD-RADS 4 in 6/94 (6.4%) and CAD-RADS 5 in 6/94 (6.4%) subjects.

In visual analysis, significant stenosis ≥ 50% in any coronary artery was present in 3/94 (3.2%) subjects. AI-analysis detected significant stenosis ≥ 50% in 7/94 (12.8%).

### Vessel level analysis

In visual analysis on vessel level, stenosis > 0% in LAD, CX and RCA was present in 32/94 (34.0%), 10/94 (10.6%) and 7/94 (7.4%), respectively.

AI-analysis detected stenosis > 0% in LAD, CX and RCA in 48/94 (51.1%), 17/94 (18.1%) and 27/94 (28.7%).

In visual analysis, significant stenosis ≥ 50% was present in 3/94 (3.2%) subjects in the LAD, and none in the CX or RCA.

AI-analysis detected significant stenosis ≥ 50% in LAD, CX and RCA in 7/94 (7.4%), 4/94 (4.3%) and 5/94 (5.3%), respectively.

### AI-based FFR-analysis

Detailed analysis of AI-based FFR-analysis is provided in Table 3. FFRai < 0.8 was detected with the AI-based model in 11/94 (11.7%) subjects, with 7/94 (7.4%) in LAD, 4/94 (4.3%) in CX, and 5/94 (5.3%) in RCA. Among 12 vessels that were labelled with CAD-RADS 4 or 5 by the AI-model, FFRai was estimated < 0.8 in 11 vessels.

**Table 3:** AI-derived FFR-analysis with corresponding CAD-RADS analysis in 94 subjects

	FFRai n (%)	AI-derived CAD-RADS n (%)	Visual CAD-RADS n (%)
FFRai > 0.8	83 (88.3)	CAD-RADS 0: 41 (43.6) CAD-RADS 1: 19 (20.2) CAD-RADS 2: 22 (23.4) CAD-RADS 3: 0 CAD-RADS 4: 1 (1.1) CAD-RADS 5: 0	CAD-RADS 0: 55 (58.5) CAD-RADS 1: 18 (19.1) CAD-RADS 2: 10 (10.6) CAD-RADS 3: 0 CAD-RADS 4: 0 CAD-RADS 5: 0
FFRai < 0.8	11 (11.7)	CAD-RADS 0: 0 CAD-RADS 1: 0 CAD-RADS 2: 0 CAD-RADS 3: 0 CAD-RADS 4: 5 (5.3) CAD-RADS 5: 6 (6.4)	CAD-RADS 0: 5 (5.3) CAD-RADS 1: 1 (1.1) CAD-RADS 2: 2 (2.1) CAD-RADS 3: 2 (2.1) CAD-RADS 4: 1 (1.1) CAD-RADS 5: 0

Variables are expressed as raw numbers; numbers in parentheses are percentages.

CAD-RADS: Coronary Artery Disease-Reporting and Data System 2.0; FFR-ai: Artificial intelligence-derived fractional flow reserve.

### Diagnostic performance

Details of diagnostic performance are provided in Table 4. On subject level, AI-dependent CAD-RADS evaluation yielded 31 true-positives, 38 true-negatives, 22 false-positives, and 3 false-negatives for the detection of any stenosis > 0%, resulting in a sensitivity, specificity, PPV, NPV, and accuracy of 91.2%, 63.3%, 58.5%, 92.7%, and 73.4%. AI-dependent evaluation for the detection of significant stenosis ≥ 50% yielded 3 true-positives, 82 true-negatives, 9 false-positives, and 0 false-negatives, resulting in a sensitivity, specificity, PPV, NPV, and accuracy of 100%, 90.1%, 25.0%, 100%, and 90.4%.

**Table 4:** Diagnostic capabilities of AI-based CAD assessment

	Sensitivity (%)	Specificity (%)	PPV (%)	NPV (%)	Accuracy (%)
*Subject level*					
Any stenosis (> 0%)	91.2 (76.3–98.1)	63.33 (49.9–75.4)	58.49 (49.9–66.6)	92.7 (80.9–97.4)	73.4 (63.3–82.0)
Significant stenosis (≥ 50%)	100.0 (29.2–100.0)	90.1 (82.1–95.4)	25.0 (15.2–38.3)	100.0 (95.6–100.0)	90.4 (82.6–95.5)
*Vessel level*					
*LAD*					
Any stenosis (> 0%)	90.6 (75.0–98.0)	69.4 (56.4–80.4)	60.42 (50.8–69.3)	93.48 (82.8–97.7)	76.6 (66.7–84.7)
Significant stenosis (≥ 50%)	100.0 (29.2–100.0)	95.60 (89.1–98.8)	42.86 (22.3–66.2)	100.00 (95.9–100.0)	95.74 (89.5–98.8)
*CX*					
Any stenosis (> 0%)	80.0 (44.4–97.5)	89.29 (80.6–95.0)	47.06 (30.8–64.0)	97.40 (91.6–99.2)	88.30 (80.0–94.0)
Significant stenosis (≥ 50%)	NA	95.74%	NA	100.0 (96.0–100.0)	NA
*RCA*					
Any stenosis (> 0%)	100.0 (59.0–100.0)	77.0 (66.8–85.4)	25.9 (19.2–34.0)	100.0 (94.6–100.0)	78.72 (69.1–86.5)
Significant stenosis (≥ 50%)	NA	94.7 (88.0–98.3)	NA	100.0 (95.9–100.0)	NA

On vessel level, AI-dependent CAD-RADS evaluation for the detection of any stenosis > 0% in LAD, CX and RCA yielded 29, 8 and 7 true-positives, 43, 75 and 67 true-negatives, 19, 9 and 20 false-positives, and 3, 2 and 0 false-negatives, respectively, resulting in a sensitivity, specificity, PPV, NPV, and accuracy of 90.6%, 69.4%, 60.4%, 93.5%, and 76.6% for LAD, 80.0%, 89.3%, 47.1%, 97.4%, and 88.3% for CX and 100%, 77.0%, 25.9%, 100%, and 78.7% for RCA, respectively.

AI-dependent CAD-RADS evaluation for the detection of significant stenosis ≥ 50% in LAD, CX and RCA yielded 3, 0 and 0 true-positives, 87, 90 and 89 true-negatives, 4, 4 and 5 false-positives, and 0 false-negatives, respectively, resulting in a sensitivity, specificity, PPV, NPV, and accuracy for the LAD of 100%, 95.6%, 42.9%, 100%, and 95.7%. Specificity and NPV for CX and RCA were 95.7% and 100%, and 94.7 and 100%, respectively.

Variables are expressed as percentages; numbers in parentheses are 95% confidence interval.

AI: artificial intelligence; CAD-RADS: Coronary Artery Disease-Reporting and Data System 2.0; CX: circumflex artery; FFR: fractional flow reserve; LAD: left anterior descending artery; NA: not applicable; NPV: negative predictive value; PPV: positive predictive value; RCA: right coronary artery.

## Discussion

This study demonstrates the potential of artificial intelligence (AI)-enhanced coronary computed tomography angiography (CCTA) as a promising non-invasive tool for detecting coronary artery disease (CAD) in asymptomatic, low-risk populations such as male marathon runners. Our findings reveal that AI-based analysis of CCTA, particularly with the integration of AI-derived fractional flow reserve (FFR), achieves high sensitivity and negative predictive value (NPV) in identifying both any CAD and significant stenosis, underscoring its utility in screening settings where traditional cardiovascular risk factors may be less prevalent. However, the study also highlights key limitations, including a lower positive predictive value (PPV) and a tendency for overestimation, reflecting the need for continued refinement of AI algorithms to improve specificity. These insights suggest that while AI-enhanced CCTA offers valuable advantages, it should be employed as a complementary tool alongside clinical expertise to optimize patient outcomes and prevent unnecessary interventions in low-risk groups.

### AI performance in low-risk populations

Our results align with previous studies demonstrating the effectiveness of AI in enhancing the diagnostic capabilities of CCTA. Similar research has reported comparable sensitivity and NPV values, reinforcing the reliability of AI-based models for non-invasive CAD detection [12, 16–18]. The AI model in our study exhibited high sensitivity (91.2% for any CAD and 100% for significant CAD), confirming its potential to detect true positives effectively, particularly in a low-risk population where early identification of subclinical disease is crucial. However, the lower PPV (25.0% for significant CAD) indicates a relatively high rate of false positives, suggesting that while the AI model is adept at recognizing disease presence, it may overestimate disease severity in some cases. This tendency towards overestimation could result in unnecessary follow-up tests or interventions, especially in low-risk populations like athletes, where false positives may lead to undue psychological stress and additional healthcare costs [6, 19, 20].

## Diagnostic accuracy and overestimation challenges

A key challenge identified in our study is the impact of common imaging artifacts, such as stepping, motion, and segmental low contrast, which might have contributed to some cases of misclassification by the AI model. While these artifacts are prevalent in routine clinical practice and were intentionally retained to assess the AI model's performance under real-world conditions, they highlight the need for further improvements. Future developments could focus on incorporating more sophisticated artifact recognition algorithms and training the AI model on datasets specifically designed to handle these challenges [13]. Furthermore, cases where misclassification occurred without apparent artifacts suggest that the model's current iteration may still have intrinsic limitations in distinguishing certain anatomical or pathological variations. Addressing these limitations will be crucial for refining AI applications in cardiovascular imaging.

### Enhancing clinical practice with AI integration

The integration of AI into clinical practice offers several advantages, including accelerated workflow, reduced inter-observer variability, and enhanced diagnostic accuracy. AI-based models are designed to complement rather than replace human interpretation, providing an automated, objective analysis that supports clinicians in making more consistent and informed decisions [18, 21, 22]. In settings with large volumes of imaging data, such as screening programs for asymptomatic athletes, AI can serve as a valuable "second reader," helping to identify early signs of subclinical disease that might otherwise be missed. This role is particularly significant in low-prevalence populations, where the primary objective is to rule out significant disease with high confidence.

## Limitations

However, several limitations must be acknowledged. First, the exclusive focus on male marathon runners limits the generalizability of our findings to other populations, including females and non-athletes. Different training regimens, genetic predispositions, and cardiovascular profiles could affect the model's performance in other groups [19, 23].

Second, the study did not utilize invasive FFR measurements as a gold standard for validating AI-derived FFR, which could affect the robustness of the conclusions. While AI-FFR provides a non-invasive option, particularly advantageous in populations where invasive testing may not be justified, discrepancies between AI-derived and invasive measurements could influence clinical decision-making [24]. Future studies should consider incorporating invasive FFR validation to strengthen the evidence base.

Additionally, the data were collected between 2012 and 2014, and advancements in imaging techniques and AI algorithms since then may influence the applicability of our findings in current practice. Future research should focus on validating these findings with more contemporary datasets and technologies.

Lastly, the need for reconstructed images to be sent to a local server for AI analysis presents practical challenges, such as data privacy concerns and workflow integration issues. Potential solutions could involve developing cloud-based AI tools or integrating AI analysis directly into existing clinical PACS systems to ensure seamless application in routine practice.

## Conclusion

In summary, AI-based CCTA shows significant potential as a non-invasive diagnostic tool for CAD, particularly in identifying significant stenosis that may require further invasive assessment, even in populations with a low prevalence of the disease. While the study demonstrates high sensitivity and diagnostic accuracy, particularly for significant stenosis, it also underscores the need for continued refinement to improve specificity. These findings suggest that AI-enhanced CCTA should be integrated as a complementary tool within clinical practice to enhance decision-making and patient management across diverse settings.

## Data Availability

The datasets generated and analyzed during the current study are not publicly available, but are available from the corresponding author on reasonable request.

## Abbreviations

AI: Artificial Intelligence
CAD: Coronary artery disease
CAD-RADS: Coronary Artery Disease-Reporting and Data System 2.0
CCTA: Coronary computed tomography angiography
CI: Confidence interval
cMPR: Curved multiplanar reconstructions
CX: Circumflex artery
FFR: Fractional flow reserve
FFRai: Artificial intelligence-derived fractional flow reserve
ICA: Invasive coronary angiography
LAD: Left anterior descending artery
MIP: Maximum intensity projection
NPV: Negative predictive value
PPV: Positive predictive value
RCA: Right coronary artery
SD: Standard deviation

## Declarations

### Competing of interest

Jean-François Paul is co-founder of Spimed-AI.
